# The association between night shift work and breast cancer risk in the Finnish twins cohort

**DOI:** 10.1007/s10654-023-00983-9

**Published:** 2023-03-25

**Authors:** Eva Schernhammer, Leonie Bogl, Christer Hublin, Susanne Strohmaier, Magda Zebrowska, Astrid Erber, Shahab Haghayegh, Kyriaki Papantoniou, Miina Ollikainen, Jaakko Kaprio

**Affiliations:** 1 Department of Epidemiology, Center for Public Health, Medical University of Vienna, Kinderspitalgasse 15, Vienna, 1090 Austria; 2 Channing Division of Network Medicine, Department of Medicine, Brigham and Women’s Hospital and Harvard Medical School, 181 Longwood Avenue, Boston, MA 02115 USA; 3 Complexity Science Hub Vienna, Josefstädter Straße 39, Vienna, 1080 Austria; 4 Institute for Molecular Medicine (FIMM), University of, Helsinki, Tukholmankatu 8, P.O. Box 20, Helsinki, 00014 Finland; 5 Finnish Institute of Occupational Health, Topeliuksenkatu 41 b, Helsinki, 00250 Finland; 6 Centre for Tropical Medicine and Global Health, Nuffield Department of Medicine, University of Oxford, New Richards Building, Old Road Campus, Roosevelt Drive, Oxford, OX3 7LG UK; 7 Minerva Foundation Institute for Medical Research, Biomedicum Helsinki 2U, Tukholmankatu 8, Helsinki, 00290 Finland

**Keywords:** Night work, Shift work, Breast cancer, Twins

## Abstract

Breast cancer is highly prevalent yet a more complete understanding of the interplay between genes and probable environmental risk factors, such as night work, remains lagging. Using a discordant twin pair design, we examined the association between night shift work and breast cancer risk, controlling for familial confounding. Shift work pattern was prospectively assessed by mailed questionnaires among 5,781 female twins from the Older Finnish Twin Cohort. Over the study period (1990–2018), 407 incident breast cancer cases were recorded using the Finnish Cancer Registry. Cox proportional hazards models were used to calculate hazard ratios (HRs) and 95% confidence intervals (CIs) adjusting for potential confounders. Within-pair co-twin analyses were employed in 57 pairs to account for potential familial confounding. Compared to women who worked days only, women with shift work that included night shifts had a 1.58-fold higher risk of breast cancer (HR = 1.58; 95%CI, 1.16–2.15, highest among the youngest women i.e. born 1950–1957, HR = 2.08; 95%CI, 1.32–3.28), whereas 2-shift workers not including night shifts, did not (HR = 0.84; 95%CI, 0.59–1.21). Women with longer sleep (average sleep duration > 8 h/night) appeared at greatest risk of breast cancer if they worked night shifts (HR = 2.91; 95%CI, 1.55–5.46; P_intx_=0.32). Results did not vary by chronotype (P_intx_=0.74). Co-twin analyses, though with limited power, suggested that night work may be associated with breast cancer risk independent of early environmental and genetic factors. These results confirm a previously described association between night shift work and breast cancer risk. Genetic influences only partially explain these associations.

## Introduction

Breast cancer remains the number one cancer among women worldwide, affecting one out of 8 women over her lifetime [1]. Genes have been identified to significantly increase a woman’s risk of breast cancer (e.g., *BRCA1* and *BRCA2*, with a 70% lifetime risk of breast cancer for the women who carry mutations in these genes [2]). Prior twin studies estimated the overall heritability of liability to breast cancer between 20% and 30% [3–5]. However, while up to 30% of breast cancer cases are linked to genetic risk, the vast majority of breast cancer cases occurs in women without known genetic mutations or familial history of breast cancer. Environmental factors such as radiation exposure or xenobiotics have previously been described to play an important role in breast carcinogenesis [6, 7]. Given that only 16% of breast cancer risk may be explained by common (early life) environmental exposures [3], studying environmental risk factors for breast cancer in adult life thus remains of central importance in any attempt to alleviate the burden of breast cancer worldwide.

Night shift work constitutes one of the most prevalent forms of circadian rhythm and sleep disruption [8, 9], with known detrimental health effects [10] for some, although not all, workers [11, 12]. In 2007 [13] and again in 2019 [14], night shift work was assessed for its carcinogenicity by the International Agency of Cancer Research (IARC), World Health Organization (WHO). It was deemed a probable (class 2 A) carcinogen in both instances, with the proclaimed need for more evidence from human studies in the most recent assessment [14].

Prior evidence suggests that common variations in circadian genes may increase the susceptibility to breast cancer among night shift workers [15–19]. Thus, the role of genetics in the association between night shift work and breast cancer risk warrants further investigation as it offers the potential for tailored interventions. We, therefore, examined the association between night shift work and breast cancer risk among the female twins of the Finnish Twin Cohort study overall and within twin-pairs. By conducting within-twin-pair analyses, which are equivalent to a nested and matched case-control analysis, with the informative pairs being those that are discordant for both breast cancer and shift work, we aimed to further explore this hypothesis. To our knowledge, this is the first study to examine these associations in a prospective study of female twins, allowing to control for potential familial confounders (genetics and shared early environment).

## Methods

### Study population

The exclusively female base population for the current analyses was nested within the Older Finnish Twin Cohort, – a long-running prospective cohort study, which started in 1975 by mailing a baseline questionnaire to all Finnish same-sex twin pairs that were born before 1958 and where both co-twins were alive in 1975 (89% response rate) [20]. Briefly, to establish the cohort, twin pairs were selected from the Central Population Registry of Finland in 1974, and twin zygosity was determined by a validated questionnaire shown to accurately classify 93% of twin pairs as monozygotic (MZ) or dizygotic (DZ) [21]. Two follow-up health surveys were conducted, mailed to all participants in 1981 and to twins born between 1930 and 1957 in 1990. The response rate to the 1981 questionnaire was 84%, and to the 1990 questionnaire 77%. The surveys contained questions on work schedules including whether the women’s work schedule comprised night work, and if so, the type of rotation; as well as sleep patterns, and chronotype. Additionally, the survey queried detailed information on socio-demographic, psychosocial, health, and lifestyle factors. Further details of the cohort have been described elsewhere [20]. Of the 11,713 female twin individuals born 1930–1957 in the cohort, the present study included all 6,804 female twin individuals who had replied to the 1990 survey. After excluding prevalent breast cancer cases (n = 43) and women residing abroad (n = 3), there were 6,758 women, among them 474 incident breast cancer cases from 1990 to 2018. After further exclusion of women who did not answer the questions on night shift work (n = 239), and those with missing data on key covariates (n = 738) including the 53 women who reported that they had never worked in their entire life (28 (53%) of these were homemakers, and 18 (34%) on disability pension), 5,781 women remained including 407 incident breast cancer cases. The study population includes 1,474 MZ and 2,568 DZ twins from pairs in which both sisters met the inclusion criteria. In addition, there were 411 MZ and 1,062 DZ twins without their co-twin and 266 twins of uncertain zygosity included in the study. The mean age (± standard deviation, SD) of the participants in the final analysis sample at the time of study entry was 43.6 years (± 7.6). The study was approved by the ethics committee of the Hjelt Institute, Faculty of Medicine, University of Helsinki. Permission for linkage of the cancer registry data was provided by the Finnish Institute for Health and Welfare, Helsinki, Finland. Informed consent was obtained from all individuals.

### Exposure assessment

Our primary exposure of interest was a woman’s self-reported (1990) shift work pattern based on current or latest work type. Rotating-shift work referred to work that rotated through morning, evening, or night shifts in either a two-shift or three-shift pattern. Shift work information was queried by assessing the respondent’s current or latest work type. The question “The present work or the work you last did (mainly) is regular day work, regular night work, two-shift work without a night shift, two-shift work with a night shift, three-shift work, or never worked”. We classified these data into 3 categories (fixed days only, rotating 2-shifts without night work, and rotating 2- or 3-shifts with night work or fixed nights). Further, night work history was also assessed in 1975 and 1981 but did not distinguish between 2-shift work with versus without night work; as well as in 2011, using the same question as posed in 1990 but only among the subset of women born in 1945–1957 who were presumably still in the workforce in 2011. We used these additional night work assessments for sensitivity analyses (1) creating a stricter definition of the reference group “never night work” (requiring never night work both at baseline 1990, as well as in earlier reports); and (2) using the 2011 night work assessment as baseline.

In addition to night/shift work exposure, information on sleep duration and quality was queried in 1990, while chronotype was assessed in 1981. Specifically, usual sleep duration over a 24-hour period, and hours of sleep needed during the night to be alert the next day [22] was assessed (nine response categories: ≤6, 6.5, 7, 7.5, 8, 8.5, 9, 9.5, and ≥ 10 h, which we collapsed into three categories: <7, 7–8, and > 8 h). Following our previous approach we considered a difference of one hour or more (usual versus needed hours of sleep duration) to indicate insufficient sleep [22]. Further, sleep quality was elicited by asking “Do you usually sleep well?” (5 response categories: well, fairly well, fairly poorly, poorly, and cannot say, which we collapsed into 3 categories: well, fairly well, and fairly poorly/poorly; “Cannot say” responses were incorporated into a missing data category for this variable). Chronotype was assessed by a question according to the Diurnal Type Scale [23] “Will you try to estimate to what extent your being ‘a morning or an evening people?” [24]. It is akin to item 19 on the Horne and Østberg morningness-eveningness questionnaire (MEQ) [25], which has previously been shown to correlate well with the overall score [25]. Based on the possible response categories, we defined four chronotypes: definite morning type, somewhat morning type, somewhat evening type, and definite evening type.

### Outcome ascertainment

Data on breast cancer incidence (ICD-10 code 174) were obtained through record linkage (using unique personal identity codes assigned to every permanent resident of Finland) to the Finnish Cancer Registry, where 100% of registered cases are histologically verified. The Finnish Cancer Registry is a nationwide database with information on all cancers diagnosed in Finland since 1953 [26]. Cancer reporting became mandatory starting in 1961. Data on emigration and vital status were obtained through linkage to the Population Register Center of Finland. Through follow-up (1990–2018; mean 25.9 years), 407 incident cases of breast cancer were accrued among women with shiftwork exposure data and with no missing data on key covariates. Information on histologic characteristics was used to explore differences by type of breast cancer (ductal versus other histological type of invasive breast cancer).

### Statistical analyses

After all exclusions, 5,781 female twin individuals who had replied to the 1990 survey that included questions on night shift work and were free of breast cancer at that time formed the base population for these analyses. Women contributed person-time from the return of the 1990 questionnaire and were censored at first report of any cancer, the date of diagnosis of breast cancer, date of death, or the end of follow-up (December 31, 2018), whichever came first. During this follow-up period (1990–2018), 5,781 women contributed 149,756 person-years at risk for breast cancer. Cox proportional hazard models (with age (in years) as the underlying time metameter) were used to calculate age-adjusted hazard ratios and 95% confidence intervals in each night work exposure category compared with the reference category. Due to the dependent nature of this sample of twin pairs, standard errors and CIs were adjusted for possible within-pair correlations using robust variance estimators. In multivariable analyses, we further adjusted for risk factors for breast cancer, including smoking status (never, occasional, former, current), current (1990) body mass index (BMI, kg/m^2^) and BMI at age 20 (queried on the 1990 questionnaire), physical activity (leisure-time metabolic equivalents METs, quintiles; assessed in 1975 and 1981), use of oral contraceptives (yes/no; assessed in 1981), alcohol consumption (number of drinks per day on average, with one standard drink defined as 12 g of alcohol, based on reported weekly or monthly consumption of beer, wines or spirits), educational status (< 6 years, 6 years, middle school, high school or more), socioeconomic status i.e. social class (upper white collar, lower white collar, skilled worker, unskilled worker, farmer, other), and zygosity (MZ, DZ, XZ). Information on age at first child-birth and number of biological children was available for women born between 1950 and 1957 (nulliparous, 1, 2, 3, 4 or more children); in sensitivity analyses restricting to these women and excluding childless women, we explored confounding by parity. Log–log plots of survival curves of the shift work exposure categories were used to verify that the proportional hazards assumption was not violated. We created indicators for missing values of categorical variables.

Secondarily, we performed a stratified analysis to explore whether chronotype or sleep quality/duration had a modifying effect on the association between rotating night shifts and breast cancer. The P value for interaction was calculated using the likelihood ratio test, which compares the models with and without the interaction term of rotating night shift work and chronotype/sleep along with the same covariates.

Lastly, a co-twin analysis to assess the association between night shift work exposure and breast cancer risk within twin pairs discordant for breast cancer was performed, though with limited power. In these analyses, we stratified Cox models on twin pairs, allowing each twin pair to have its own baseline hazard [27]. This serves as a powerful approach to account for potential familial confounding (genetics and shared family environment) when assessing twins discordant for night shift work and breast cancer outcomes. All statistical analyses were performed using Stata version 17.0 (Stata Corporation, College Station, TX, USA).

## Results

Participant characteristics at baseline are presented in Table 1. The average age of women who worked days only (43.6 years) did not differ from those who worked 2-shifts (44.1 years) or 3-shifts or night work (43.9 years).

**Table 1:** Baseline characteristics by night work pattern, Older Finnish Twin Cohort, 1990.*

	Shift work in 1990			
Characteristics	**Daywork**	**2-shift w/o night** ^*^	**Night and 3-shift** ^*^	**Overall**
	N = 4,640	N = 614	N = 464	N = 5,718
Age at response to 1990 survey	43.6 (7.6)	44.1 (7.6)	43.9 (7.5)	43.7 (7.6)
BMI in 1990	23.8 (3.8)	24.3 (4.2)	23.9 (3.7)	23.8 (3.9)
BMI at age 20	20.7 (2.3)	21.2 (2.8)	21.1 (2.4)	20.8 (2.4)
Number of biological children	1.8 (1.4)	1.8 (1.2)	2.0 (1.5)	1.8 (1.4)
Education in 1981				
<6 yrs	25 (0.5%)	4 (0.6%)	4 (0.8%)	33 (0.6%)
6 yrs	1,434 (30.6%)	260 (41.8%)	117 (24.8%)	1,811 (31.3%)
Middle school	2,181 (46.5%)	261 (42.0%)	264 (56.1%)	2,706 (46.8%)
Highschool or more	905 (19.3%)	76 (12.2%)	68 (14.4%)	1,049 (18.1%)
Missing	143 (3.1%)	21 (3.4%)	18 (3.8%)	182 (3.1%)
Leisure time physical activity 1981				
Sedentary	510 (11.2%)	67 (11.1%)	51 (11.2%)	628 (11.2%)
Other	3,639 (80.0%)	486 (80.7%)	352 (77.5%)	4,477 (79.9%)
Conditioner	401 (8.8%)	49 (8.1%)	51 (11.2%)	501 (8.9%)
Employed in 1990				
No	1,231 (26.7%)	130 (21.1%)	110 (23.6%)	1,471 (25.8%)
Yes	3,381 (73.3%)	486 (78.9%)	357 (76.4%)	4,224 (74.2%)
Cigarette smoking status 1990				
Never	2,726 (58.1%)	348 (55.9%)	278 (59.0%)	3,352 (58.0%)
Occasional	144 (3.1%)	21 (3.4%)	13 (2.8%)	178 (3.1%)
Former	858 (18.3%)	107 (17.2%)	78 (16.6%)	1043 (18.0%)
Current	960 (20.5%)	146 (23.5%)	102 (21.7%)	1208 (20.9%)
Heavy drinking occasions at least monthly 1990				
No	4,110 (88.3%)	526 (85.1%)	418 (89.9%)	5,054 (88.1%)
Yes	546 (11.7%)	92 (14.9%)	47 (10.1%)	685 (11.9%)
Sleep duration in 1990				
Short	686 (14.7%)	97 (15.7%)	103 (22.0%)	886 (15.4%)
Medium	3,216 (68.8%)	423 (68.3%)	282 (60.1%)	3,921 (68.0%)
Long	774 (16.6%)	99 (16.0%)	84 (17.9%)	957 (16.6%)
Sleep quality in 1990				
Good	2,485 (53.0%)	289 (46.5%)	248 (52.7%)	3,022 (52.3%)
Fairly good	1,354 (28.9%)	194 (31.2%)	124 (26.3%)	1,672 (28.9%)
Fairly poor/poor	838 (17.9%)	138 (22.2%)	97 (20.6%)	1,073 (18.6%)
Cannot say or missing	11 (0.2%)	1 (0.2%)	2 (0.4%)	14 (0.2%)
Ever used oral contraceptives (OCs) (1981)				
Never	1,803 (43.3%)	256 (48.7%)	171 (42.9%)	2,230 (43.8%)
Ever	2,362 (56.7%)	270 (51.3%)	228 (57.1%)	2,860 (56.2%)
Social class 1975				
Upper white-collar	257 (5.5%)	8 (1.3%)	7 (1.5%)	272 (4.7%)
Lower white-collar	1,688 (36.0%)	139 (22.3%)	191 (40.6%)	2,018 (34.9%)
Skilled workers	1,388 (29.6%)	292 (46.9%)	145 (30.8%)	1,825 (31.6%)
Unskilled workers	493 (10.5%)	104 (16.7%)	52 (11.0%)	649 (11.2%)
Farmers	241 (5.1%)	19 (3.1%)	8 (1.7%)	268 (4.6%)
Other (students, retired, unknown)	621 (13.2%)	60 (9.6%)	68 (14.4%)	749 (13.0%)

* Data are presented as means and standard deviations, or percentages. Shift work categorizations are based on the question “The present work or the work you last did (mainly) is regular day work, regular night work, two-shift work without a night shift, two-shift work with a night shift, three-shift work, or never worked”

The relationship between 3-shift night work and breast cancer risk is shown in Table 2. In age-adjusted analyses, we observed a significantly increased risk of breast cancer among the women working 3-shift night work (HR = 1.52, 95%CI 1.12–2.06), compared to those who had worked days only. This risk became only slightly stronger after further adjustment for potential confounders: Women on the 3-shift night work schedule had a 1.58-fold increased risk of breast cancer, compared to those working days only (multi-variable adjusted HR = 1.58, 95%CI 1.16–2.15). By contrast, women working 2-shifts without nights did not experience an elevated risk of breast cancer (multivariable-adjusted HR = 0.84; 95%CI, 0.59–1.21). In sensitivity analyses restricting to women for whom parity information was available, and excluding childless women, estimates remained largely unchanged. Further sensitivity analyses using a reference group of never night work (i.e. using also earlier reports on night work status from 1975 to 1981) and with shorter follow-up starting in 2011 did not produce any noteworthy differences in results (data not shown).

**Table 2:** Shift work, chronotype, sleep duration, sleep quality, and breast cancer risk (HR and 95% CI) among 5,781 women, of whom 407 had incident breast cancer in the Older Finnish Twin Cohort, 1990–2018

		Breast cancer incidence		
	Person-years	No. events	Age-adjusted	Fully adjusted
***OVERALL***:				
**Shift work pattern** ^**%**^				
Days only	121,532	325	1.0 (Ref.)	1.0 (Ref.)
2-shifts without nights	16,278	33	0.75 (0.52–1.07)	0.84 (0.59–1.21)
3-shifts or nights only	11,947	49	1.52 (1.12–2.06)	1.58 (1.16–2.15)
***STRATIFIEDANALYSES***:				
**Morning chronotypes** ^**#**^				
Days only	64,567	182	1.0 (Ref.)	1.0 (Ref.)
2-shifts without nights	7,890	18	0.79 (0.49–1.29)	0.91 (0.55–1.47)
3-shifts or nights only	5,916	24	1.43 (0.92–2.22)	1.46 (0.93–2.28)
**Evening chronotypes**				
Days only	52,811	140	1.0 (Ref.)	1.0 (Ref.)
2-shifts without nights	7,770	13	0.62 (0.35–1.10)	0.73 (0.41–1.31)
3-shifts or nights only	5,575	22	1.47 (0.94–2.31)	1.56 (0-99-2.46)
*P for interaction*				*0.993*
**Short sleep duration (< 7 h)** ^*^				
Days only	17,613	50	1.0 (Ref.)	1.0 (Ref.)
2-shifts without nights	2,507	3	0.42 (0.13–1.35)	0.44 (0.13–1.50)
3-shifts or nights only	2,587	8	1.09 (0.51–2.32)	1.08 (0.44–2.41)
**Medium sleep duration (7–8 h)**				
Days only	83,423	229	1.0 (Ref.)	1.0 (Ref.)
2-shifts without nights	11,203	24	0.77 (0.51–1.17)	0.86 (0.57–1.32)
3-shifts or nights only	7,170	27	1.34 (0.89–2.03)	1.40 (0.92–2.13)
**Long sleep duration (> 8 h)**				
Days only	20,171	45	1.0 (Ref.)	1.0 (Ref.)
2-shifts without nights	2,487	6	1.07 (0.46–2.52)	1.28 (0.50–3.25)
3-shifts or nights only	2,145	13	2.78 (1.51–5.03)	2.91 (1.55–5.46)
*P for interaction*				*0.351*

* 17 women/2 cases had missing data on sleep duration

^#^ 203 women/8 cases had missing data on diurnal type from 1981; Chronotype had no main effect on breast cancer risk (multivariable adjusted HR = 0.92, 95%CI, 0.75–1.12)

^**%**^ Shift work categorizations are based on the question “The present work or the work you last did (mainly) is regular day work, regular night work, two-shift work without a night shift, two-shift work with a night shift, three-shift work, or never worked”

In analyses stratified by chronotype, we found that the positive association between 3-shift night work and breast cancer risk was similar, regardless of chronotype or sleep duration (short, medium, long; all P_intx_>0.05; Table 2). Of note, though based on only 13 breast cancer cases, among the women with on average longer sleep duration (more than 8 h), 3-shift/night work was more strongly associated with breast cancer risk (HR = 2.91, 95% CI 1.55–5.46), whereas this was not the case among women with 7–8 h of sleep (HR = 1.40; 95%CI, 0.92–2.13), or women sleeping on average less than 7 h (HR = 1.08, 95% CI 0.44–2.41; P_intx_=0.32).

The majority (n = 293; 72%) of all breast cancers were ductal carcinomas, with 71 lobular and the remaining tumors of varying histology. Due to small case numbers, we were not able to explore differences in risks by histology. However, restricting to the ductal breast cancer cases only did not alter our estimates substantially (data not shown).

**Fig. 1:**
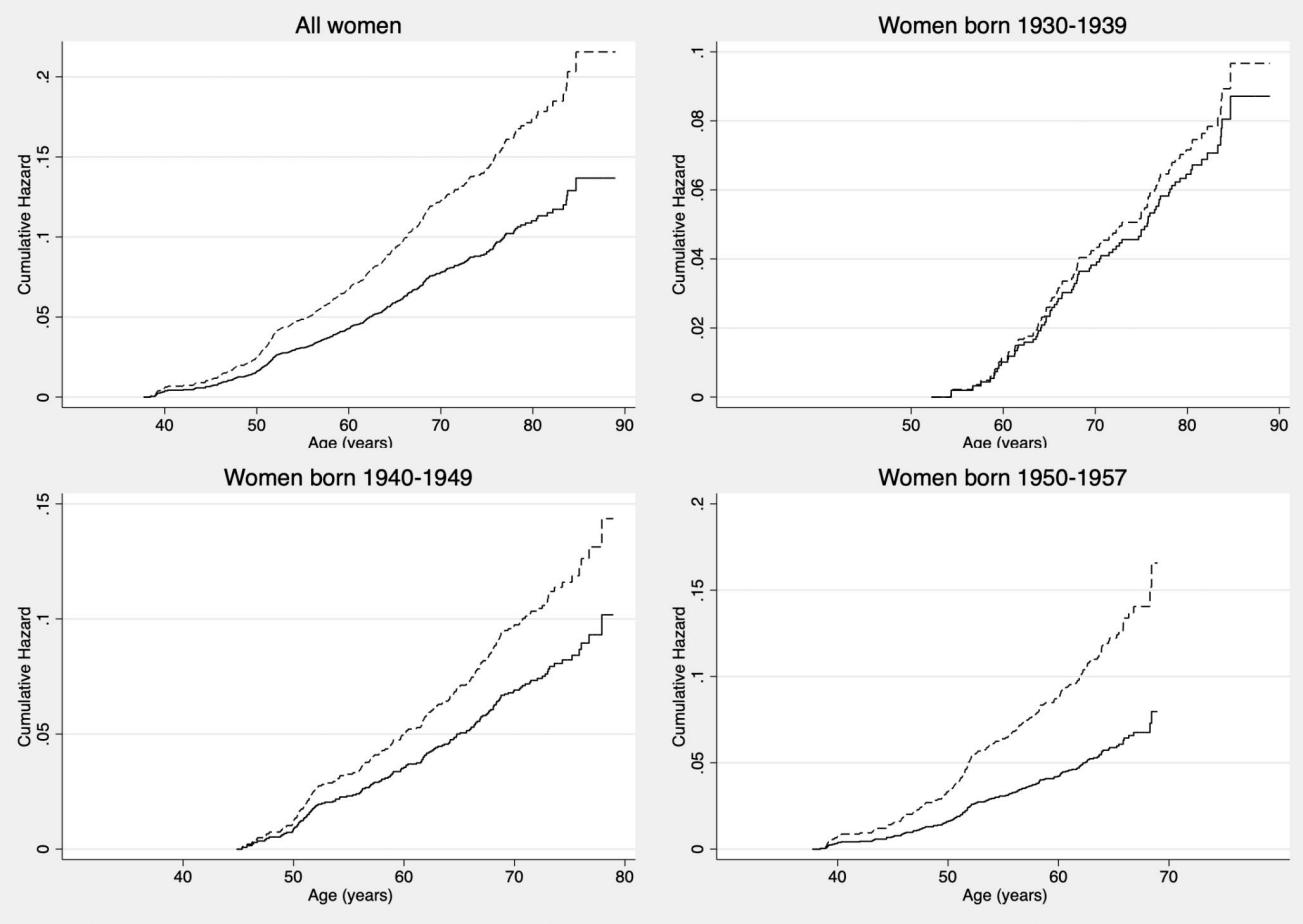
Cumulative hazard incidence of breast cancer among women working night/shiftwork (dashed line) vs. women working days only (solid line), overall and by birth decade. Solid line: day work; dashed line: night/shift work; Top left: All women; Top right: Women born 1930–1939; Bottom left: Women born 1940–1949; Bottom right: Women born 1950–1957

Coincidentally i.e., without prior hypothesis but as part of our verification of the proportionality assumption (which was not violated, as stated earlier), we observed considerable differences in the risk of breast cancer among night workers depending on the women’s birth decade (Fig. 1). Specifically, women working night shifts who were born in the 1930s (1930–1939) had no increased risk of breast cancer (HR = 1.11; 95%CI, 0.50–2.48), women born in the 1940s (1940–1949) had a 41% non-significantly increased risk (HR = 1.41; 95%CI, 0.89–2.33), and the women born in the 1950s (1950–1957) who worked night shifts had a significantly elevated risk of breast cancer (HR = 2.08; 95%CI, 1.32–3.28), compared to day working women. Though parity is unlikely to explain this difference because it peaked in Finland in post-war year 1947, following low fertility in the 1930s [28], we further adjusted for parity in sensitivity analyses and results remained largely unchanged for women born in the 1950s.

In a co-twin analysis to assess the association between night shift work exposures and breast cancer risk within twin pairs discordant for breast cancer, we observed 25 pairs where the breast cancer case worked night shift (n = 25) and co-twin sister did not (of these co-twins, 20 were day workers and five were 2-shift workers); and 17 pairs where the healthy sister worked night shifts and the breast cancer case did not (14 day workers and three 2-shift workers). For all pairs where the numbers were large enough to allow analysis, the conditional logistic regression for matched pair data (which ignores time to event and censoring) estimated a 41% increased but statistically non-significant risk (adjusted OR = 1.41, 95%CI, 0.75–2.64). The stratified Cox HR was slightly smaller (HR = 1.34, 95%CI, 0.73–2.45). After adjustment for all covariates, the estimates did not change (adjusted OR = 1.35, 95%CI, 0.63–2.89, adjusted HR = 1.39, 0.66–2.90).

The pairwise estimates in these analyses were very similar to those from the analyses on individuals, suggesting a higher risk of breast cancer among night workers even after accounting for potential familial confounding (genetics and shared family environment). There were also three twin pairs concordant for night work despite discordance for breast cancer (two of them DZ twins, and 1 of them MZ).

## Discussion

To our knowledge, no prior study on the impact of night work on breast cancer risk among twins exists. Consistent with previous reports among singletons, we found an elevated risk of breast cancer among women working night shifts in a large, prospective, population-based cohort study of Finnish female twins. Specifically, women who worked two or three shifts involving night work, or night shifts only, had a significantly higher risk of breast cancer than women working days only, either as day work or two-shift work. While there was no interaction between night work and sleep or chronotype, compared to day workers, we observed a significantly increased risk of breast cancer among women with longer sleep duration, i.e., more than 8 h of sleep on average, but not among the women with 8 or fewer hours of average sleep. Neither sleep duration, nor sleep quality or chronotype were individually associated with breast cancer risk in this cohort. Taken together, our data are confirmatory of earlier reports of an increased breast cancer risk among night workers, and they provide unique suggestive evidence for additional effects of night work beyond an individual’s genetic risks.

Night work and its association with cancer risk has been extensively studied in prior analyses and was evaluated by large consortia in conjunction with the existing evidence from animal studies and mechanistic studies [13, 29, 30]. Overall, considering all cancer endpoints, the evidence tended to be strongest for breast cancer [31] given the higher number of existing studies, likely owed to the high prevalence of breast cancer in women and thus more powerful analyses possible when using this endpoint. A recent summary (Monograph 124, Carcinogenicity of night shift work [30]) concluded that the most powerful human evidence on night shift work and cancer risk comes from case-control studies [32], with their more detailed shift schedule assessments compared to large-scale cohort studies such as the Nurses’ Health Study [33]. Given the greater potential for non-differential misclassification in cohort studies [34], where night work questions were posed several decades ago without the appreciation of the importance of any measures of duration and intensity of night work, compared to case-control studies, estimates from cohort studies tend to miss existing associations due to their inherent bias towards the null. In light of this, a positive association as observed in our study is very reassuring, as the unbiased results are likely higher than demonstrated. Therefore, our results are much in line with the existing evidence.

Mechanistically, multiple pathways have now been demonstrated in support of a night work – cancer association [35], most of them based on the central role of the cellular circadian clock in coordinating cell division and DNA repair. Observations from animal and experimental studies support that disrupted cellular clocks accelerate cancer cell growth [35]. These mechanisms center around the exposure to light at night experienced by night workers, and the resulting disruption of their circadian system including melatonin levels, but also the inflammatory and immune system and hormonal pathways [36]. Night work may also affect the epigenetic regulation of key clock genes and other relevant genes, thus possibly further contributing to a higher breast cancer risk among night workers [37].

A second finding of interest in our analyses pertains to the results on discordant twin pairs. Though underpowered, these suggest an effect of night work above and beyond familial and genetic risks. That the association between night work and breast cancer risk was slightly attenuated in the co-twin analyses discordant on night work and breast cancer outcome, compared to the main effects (HR around 1.5 versus here, ~ 1.4) may suggest that the main effects could at least partially have been explained by an unaccounted for shared genetic or environmental factor, and that the possibility of a factor influencing both night work and breast cancer should be further explored. While no evidence exists to support the heritability of the propensity to work night shifts, an earlier report from the powerful Nordic Twin Study of Cancer (NorTwinCan) estimated the heritability of liability to breast cancer to be about 20 to 28% [3]. Alternatively, our findings of a somewhat weaker association between night work and breast cancer risk among the discordant twin pairs may be owed to the smaller numbers and thus lower power with greater uncertainty in the effect estimates. Adjustment for multiple covariates did not changes the estimates compared to the analysis of discordant pairs (who are already matched on age and multiple childhood and adolescent experiences and exposures.).

Interestingly, in our study, the night workers with longer sleep duration appeared to be at highest risk of breast cancer though case numbers were small in this stratum. Examinations of the interaction between night shift work and sleep duration and their association with cancer risk have only recently been emerging. They highlight the phenomenon of shift work disorder, i.e. a night shift worker who develops a set of symptoms lasting for at least 3 months in response to their night schedules. Symptoms foremost include sleep problems in the form of insomnia; but also excessive sleepiness and to some extent sleep duration [38]. Several studies though not entirely consistently [39] found no effect of sleep duration on prostate cancer risk in night shift workers [40–43]. For breast cancer, only one other study has been published, to date, reporting that night workers had a higher risk of breast cancer particularly the women with shorter or longer sleep duration, compared to women with > 6 but < 9 h of sleep [44]. More research is warranted to further examine the importance of sleep duration in these associations. If substantiated, sleep duration could emerge as an early predictor for individual disease risk subsequent to night shift work exposure.

Contrary to a previous studies examining the association between chronotype, work type, and prostate (42) or breast cancer risk [45], we did not observe effect modification by chronotype in our analyses. However, Dickerman et al. (42) were not able to examine the interaction between chronotype and night work specifically, given their very small case numbers (only 2 prostate cancer cases in these analyses). In general, evidence is increasingly supportive of an additive effect of chronotype and night work, where health risks may be different among individuals whose work time is mismatched with their chronotype [46] (e.g., a night worker with a morning preference), though evidence from human studies is still scarce and inconsistent to draw any firm conclusions (42, 46, 48–52). However, chronotype does not only have a moderately strong genetic [24, 52] but likely also an important early life environmental component; and the discordant twin study design allowed us to fully account for these factors.

Lastly, our observation of a birth cohort effect in our results deserves note. We are not aware of any earlier reports that have examined whether their results of an association between night work and cancer risk differed by birth cohort of the study participants. However, the inherently longer follow-up among the older birth cohorts will likely have increased the post-retirement time for these women; and we have previously described that night-work associated breast cancer risk may wane once a woman stops working night shifts i.e. in retirement [33]. Another potential explanation for this interesting observation might relate to differences in parity (an important risk factor for breast cancer) over time – though the time trends in Finland (i.e. parity peaking in 1947) are contrary to what one would expect for it to serve as an explanation for our observed differences; moreover, in sensitivity analyses adjusting for parity results in the youngest birth cohort remained largely unchanged. Similarly, differential usage of oral contraceptives might have contributed to this observation [53] though adjustment for this variable did not materially alter our findings. Alternatively, changes in the work environment or intensity of night work may have occurred over time, with more frequent night shifts or less sleep possible during night shifts in the later decades of our study sample; though we have no way to verify this speculation. Given the increasing breast cancer risk among night working women in the later decades, further studies appear warranted to explore whether this trend continues past the time periods observed in our study.

Our study has several limitations of note. We were not able to adjust for a few important breast cancer risk factors including menopausal status, postmenopausal hormone use, and age at first birth. However, information on these variables was available in a smaller subset of the women, which allowed us to conduct sensitivity analyses; reassuringly, results did not change by much in these analyses. Nonetheless, as in any observational study, the potential for residual and uncontrolled confounding by e.g., other occupational factors, remains. Further, important differences in the associations by ethnicity/culture have previously been noted [54]; given that all twins in our study were of European ancestry, our results may therefore not be generalizable to other populations. Another limitation of note pertains to the self-reported nature of our night shift work assessment and lack of detailed exposure metrics (e.g., duration and intensity of night work); however, with additional night work assessments over time we were able to conduct sensitivity analyses albeit in smaller subsamples, but largely confirming our main results of a higher risk of breast cancer among night workers. Nonetheless, non-differential misclassification cannot be ruled out, suggesting that our results may have been attenuated owing to this bias.

Some strengths of our study also deserve mention. First, the follow-up time of up to 28 years, from 1990 to 2018, is long enough for a chronic disease like breast cancer to occur. Second, as twins do not differ from singletons in e.g., cancer incidence [55] and sleep lengths [56], the national representative sample allows for tentative generalization of the findings to the general population. Moreover, the use of nationwide reliable population register data represents another strength of our analyses.

In conclusion, in this prospective, population-based cohort study of Finnish female twins, we found a significant association between night work and breast cancer risk, with some suggestively stronger effects in the subgroup of women with longer sleep duration requiring confirmation. Results from discordant twin pair analyses addressing the shared genetic and environmental risk factors for breast cancer suggest that night work may have carcinogenic effects above and beyond familial risks.

## Data Availability

There is no new data associated with this article. The existing data is available through the Institute for Molecular Medicine Finland (FIMM), Data Access Committee (DAC) (fimmdac@helsinki.fi) for authorized researchers who have IRB/ethics approval and an institutionally approved study plan. To ensure the protection of privacy and compliance with national data protection legislation, a data use/transfer agreement is needed, the content and specific clauses of which will depend on the nature of the requested data.
